# Prevalence and clinical implications of germline predisposition gene mutations in patients with acute myeloid leukemia

**DOI:** 10.1038/s41598-020-71386-z

**Published:** 2020-08-31

**Authors:** Borahm Kim, Woobin Yun, Seung-Tae Lee, Jong Rok Choi, Keon Hee Yoo, Hong Hoe Koo, Chul Won Jung, Sun Hee Kim

**Affiliations:** 1 Department of Laboratory Medicine, Korea University College of Medicine, Seoul, Korea; 2 Department of Laboratory Medicine, Yonsei University College of Medicine, 50 Yonsei-ro, Seodaemun-gu, Seoul, 03722 Korea; 3 Brain Korea 21 PLUS Project for Medical Science, Yonsei University, Seoul, Korea; 4 Department of Pediatrics, Samsung Medical Center, Sungkyunkwan University School of Medicine, Seoul, Korea; 5 Department of Internal Medicine, Samsung Medical Center, Sungkyunkwan University School of Medicine, Seoul, Korea; 6 Department of Laboratory Medicine and Genetics, Samsung Medical Center, Sungkyunkwan University School of Medicine, 81 Ilwon-ro, Gangnam-gu, Seoul, 06351 Korea

**Keywords:** Genetics, Cancer genomics, Haematological cancer

## Abstract

Acute myeloid leukemia (AML) is one of the most common types of leukemia. With the recent advances in sequencing technology and the growing body of knowledge on the genetics of AML, there is increasing concern about cancer predisposing germline mutations as well as somatic mutations. As familial cases sharing germline mutations are constantly reported, germline predisposition gene mutations in patients with AML are gaining attention. We performed genomic sequencing of Korean patients diagnosed with AML to identify the prevalence and characteristics of germline predisposition mutations. Among 180 patients, germline predisposition mutations were identified in 13 patients (13/180, 7.2%, eight adults and five children). Germline mutations of *BLM*,* BRCA1*,* BRCA2*,* CTC1*,* DDX41*,* ERCC4*,* ERCC6*,* FANCI*,* FANCM*,* PALB2*, and *SBDS* were identified. Most of the mutations are in genes involved in DNA repair and genomic stability maintenance. Patients harboring germline mutations tended to have earlier onset of AML (*p* = 0.005), however, the presence of germline mutations did not showed significant association with other clinical characteristics or treatment outcome. Since each mutation was rare, further study with a larger number of cases would be needed to establish the effect of the mutations.

## Introduction

With the recent advances in sequencing technology and the growing body of knowledge on the genetics of AML, there is increasing concern about cancer predisposing germline mutations as well as somatic mutations. It has been widely recognized that not only somatic mutations in cancer tissue, but also germline gene mutations can affect disease characteristics, progress, and prognosis, and In cases with solid cancers such as breast cancer, the significance of germline mutation had been already recognized and changes in treatment and genetic counseling according to the presence of those mutations had been settled down in clinical practice. Similarly, the category myeloid neoplasm with germline predisposition mutations was included in the WHO classification in 2016^1^.

A number of cases of familial leukemia with these mutations was reported, mainly in relatively well-recognized genes such as *DDX41*, *CEBPA*, and *RUNX1*^2–4^. However, these studies focused on specific ethnic groups, and data regarding other ethnicities are lacking. Furthermore, for patients with AML, hematopoietic stem cell transplantation (HSCT) is frequently performed. Germline predisposition mutations could be a significant issue in the setting of HSCT, where most donors are family members, and a higher probability of shared mutations is expected. As in the case of *BRCA1/2* gene mutation, there may be an increased risk of other cancers, and patients and family members having the same mutation should be involved in a surveillance program. Therefore, there is a growing need for basic information about frequency and types of germline predisposition gene mutations.

In this context, we assessed the prevalence of germline predisposition gene mutations and identified the clinical characteristics of mutation carriers among Korean patients with AML using genomic sequencing and we correlated the mutation status with treatment outcome, to provide a broader insight into AML biology and patient care.

## Methods

### Patients

Bone marrow aspirates of 180 patients diagnosed with AML in our center between 2013 and 2017 were obtained after receiving informed consent for genetic study from each patient. This study was approved by the Samsung Medical Center institutional review board (2017-04-027) and was conducted in accordance with the tenets of the Declaration of Helsinki.

### Genomic sequencing

A total of 139 patients was evaluated by whole exome sequencing (WES) and 41 patients were by gene panel sequencing. NGS testing was performed with initial diagnostic samples. Clinical characteristics of patients were in Supplemental Table 1. For WES, libraries were prepared using the Twist Human Core Exome Kit (Twist Bioscience, San Francisco, CA, USA) and sequenced on a NovaSeq system (Illumina, San Diego, CA, USA). For gene panel sequencing, gene panel containing 215 genes (Supplemental Table 2) associated with hematologic malignancy or germline cancer predisposition was used and NextSeq 550 instrument (Illumina) was used. Detailed sequencing methods and bioinformatics analysis were in Supplemental Methods.

### Interpretation of variants

The following databases were used for variant annotation: Online Mendelian Inheritance in Man (OMIM), Human Gene Mutation Database (HGMD), ClinVar, dbSNP, 1,000 Genome, he Genome Aggregation Database (gnomAD), Exome Sequencing Project (ESP), and the Korean Reference Genome Database (KRGDB). The pathogenicity of missense variants was predicted using five in silico prediction algorithms, including Sorting Tolerant from Intolerant (SIFT), Polymorphism Phenotyping v2 (PolyPhen-2), MutationTaster, MutationAssessor, and Functional Analysis through Hidden Markov Models (FATHMM), implemented in dbNSFP. Effects on splicing were predicted using SPIDEX, and dbscSNV.

We first established a set of genes known to be associated with AML predisposition according to WHO classification^5^, and variants of those genes were prioritized. Variants were further classified according to American College of Medical Genetics (ACMG) guideline^6^. For PM2 score, global population frequency cut off < 0.00001 for dominant disease and < 0.0001 for recessive disease were applied. For PP3 score, the agreement of at least five prediction tools was applied.

### Germline confirmation test

Suspected variants in germline predisposition genes were further confirmed with bone marrow specimens were collected when the patients were in complete remission. Sanger sequencing was performed using custom primers and the BigDye Terminator Cycle Sequencing Ready Reaction Kit on an ABI Prism 3730 Genetic Analyzer (Applied Biosystems, Foster City, CA, USA). Because bone marrow was used for germline mutation testing, possibility of confounding due to factors including residual tumor and clonal hematopoiesis cannot be ruled out. Interpretation of Sanger sequencing results was proceeded under recognition of the limitation.

### Statistical analysis

To compare the outcome according to molecular characteristics, Fisher’s exact test and logistic regression analysis were utilized, with single or multiple variables. Variables included in multiple logistic levels were chosen by adapting stepwise as a variable selection method. Statistical analyses were computed using R. *p* values < 0.05 were considered statistically significant.

## Results/discussion

For gene panel sequencing, diagnostic exome sequencing, median sequence coverage was 1626x, with an average of genotype quality score 98. For whole exome sequencing, median sequence coverage was 182x, with an average of genotype quality score 54.

### Genes involved in DNA repair or maintenance of genomic stability were frequently mutated

Among 180 patients, 13 (13/180, 7.2%) showed pathogenic mutations in germline predisposition genes (Table 1). Most of the identified germline predisposition gene mutations were in genes involved in DNA repair or maintenance of genomic stability, which were associated with inherited bone marrow failure syndromes including Fanconi anemia (FA), dyskeratosis congenita (DC), or Shwachman–Diamond syndrome (SDS).

**Table 1:** Identified germline predisposition gene mutations.

ID	Age	Gene	OMIM disease	Variant^a^	VAF	Depth	Type	ClinVar	ACMG classification	Population frequency^b^ Esat Asian global		Accompanied somatic mutations^c^
P1561	30	*BRCA1* *NM_007294.3*	Familial breast-ovarian cancer 1, 604,370, AD	c.2433delC p.Lys812ArgfsTer3	0.54	127	Frameshift	Pathogenic	Pathogenic (PVS1, PM1, PM2, PP3, PP5)	0.00005437	0.000007981	*KMT2A-MLLT3*
P3065	62	*BRCA2* *NM_000059.3*	Familial breast-ovarian cancer 2, 612,555, AD Fanconi anemia, complementation group D1, 605,724, AR	c.2798_2799delCA p.Thr933ArgfsTer2	0.39	137	Frameshift	Pathogenic	Pathogenic (PVS1, PM2, PP5)	0	0	*IDH2*,* DNMT3A*
P1052	45	*PALB2* *NM_024675.3*	Fanconi anemia, complementation group N, 610,832 Breast cancer, susceptibility to, 114,480, AD Pancreatic cancer, susceptibility to	exon 11 deletion	0.51	1,050	Frameshift	Not reported	Likely pathogenic (PVS1, PM2)	0	0	*DNMT3A*,* FLT3* ITD, *KMT2A* PTD
P1527	7	*FANCM* *NM_020937.2*	Premature ovarian failure 15, 618,096, AR Spermatogenic failure 28, 618,086, AR	c.1456C > T p.Arg486Ter	0.54	254	Nonsense	Not reported	Likely pathogenic (PVS1, PM2)	0	0	*BRCA2*
P2912	58	*FANCI* *NM_001113378.1*	Fanconi anemia, complementation group I, 609,053	c.158-2A > G	0.54	113	Splice site	Likely pathogenic	Likely pathogenic (PVS1, PM2)	0.00005437	0.000003977	*RUNX1-RUNX1T1*
P1415	3	*FANCI* *NM_001113378.1*	Fanconi anemia, complementation group I, 609,053	c.158-2A > G	0.6	115	Splice site	Likely pathogenic	Likely pathogenic (PVS1, PM2)	0.00005437	0.000003977	*KIT*
P2605	5	*ERCC4* *NM_005236.2*	Fanconi anemia, complementation group Q, 615,272, AR	c.458G > A p.Arg153His	0.48	2,311	Missense	Not reported	Likely pathogenic (PM1, PM2, PM5, PP3)	0.0001631	0.00002386	*FLT3*,* FLT3* ITD, *PML-RARA*
P0655	0	*SBDS* *NM_016038.2*	Aplastic anemia, susceptibility to, 609,135 Shwachman–Diamond syndrome, 260,400, AR	c.258 + 2T > C	0.53	90	Splice site	Pathogenic	Pathogenic (PVS1, PM2, PP3)	0.009412	0.003879	*GATA1*
P3036	9	*SBDS* *NM_016038.2*	Aplastic anemia, susceptibility to, 609,135 Shwachman–Diamond syndrome, 260,400, AR	c.258 + 2T > C	0.51	143	Splice site	Pathogenic	Pathogenic (PVS1, PM2, PP3)	0.009412	0.003879	*NRAS*, *KMT2A-MLLT3*
P0654	58	*ERCC6* *NM_000124.3*	Cerebro-oculo-facio-skeletal syndrome 1, 214,150, AR Cockayne syndrome, type B, 133,540, AR Lung cancer, susceptibility to, 211,980, AR	c.2169 + 1G > A	0.54	109	Splice site	Likely pathogenic	Likely pathogenic (PVS1, PM2)	0	0	*CEBPA*,* GATA2*, *KMT2D*,* SH2B3*
P1256	40	*BLM* *NM_000057.4*	Bloom syndrome, 210,900, AR	c.320dupT p.Leu107PhefsTer36	0.45	114	Frameshift	Pathogenic	Pathogenic (PSV1, PM2)	0.0001632	0.00001194	
P1562	42	*CTC1* *NM_025099.5*	Cerebroretinal microangiopathy with calcifications and cysts, 612,199, AR	c.19C > T p.Gln7Ter	0.48	1,424	Nonsense	Not reported	Likely pathogenic (PVS1, PM2)	0.00002952	0.00001679	*CEBPA*,* DNMT3A*, *BCORL1*,* IDH2*
P1788	44	*DDX41* *NM_016222.2*	Familial myeloproliferative/lymphoproliferative neoplasms, susceptibility to, 616,871, AD	c.1496dupC p.Ala500CysfsTer9	0.42	241	Frameshift	Not reported	Likely pathogenic (PVS1, PM2)	0.0001087	0.000007956	

*VAF* variant allele fraction, *ITD* internal tandem duplication, *PTD* partial tandem duplication, *VOUS* a variant of unknown significance.

^a^Variants were confirmed as germline origin using bone marrow aspirate in complete remission.

^b^Population frequencies from gnomAD (https://gnomad.broadinstitute.org/).

^c^Tier 1 or Tier 2 somatic variants according to published guideline (Li et al., J Mol Diagn 2017, PMID 27993330) were presented.

Eight of the 13 germline mutations identified were in six FA genes: *FANCD1* (*BRCA2*), *FANCI*, *FANCM*,* FANCN* (*PALB2*), *FANCQ (ERCC4)*, and *FANCS* (*BRCA1*). FA genes are involved in the FA signaling pathway, which is crucial in the DNA damage response. Constant exposure to endogenous and exogenous genotoxic agents can jeopardize genomic stability when the DNA damage response is compromised^7^. FA is a rare genetic disorder often accompanied by numerous other conditions, including early age of onset of symptoms, multiorgan congenital defects, bone marrow failure leading to pancytopenia, and predisposition to hematological and non-hematological malignancies^8^. Monoallelic mutation carriers who had no apparent signs, were known to have increased risk for cancer^9–11^. The protein products of these six genes are components of the DNA damage response system and participate in various cellular processes. Genomic instability resulting from defective function of those proteins can be associated with increased cancer risk. However, elucidation of the function of each component and the consequences of deficiencies of each product have yet to be determined.

In addition, *BRCA1*/2, and *PALB2* are not only associated with FA and predisposition for AML, but are also widely known as important risk factors for breast and ovarian cancer. Although monoallelic mutation carriers do not develop FA, monoallelic mutations in *BRCA1*/2, or *PALB2* are associated with increased risk of specific cancers, e.g., breast cancer. Recent National comprehensive cancer network (NCCN) guidelines suggest that carriers of those mutations should be included in the surveillance program and should consider preventive actions (https://www.nccn.org/professionals/physician_gls/pdf/genetics_screening.pdf). The same principle should hold for the patient with AML with germline predisposition mutations.

Besides, *BLM* is associated with Bloom syndrome, a rare chromosomal instability disorder characterized by growth retardation, immunodeficiency, and a wide spectrum of cancers^12^. *BLM* mutation is known to be associated with cancers^13^. *ERCC6* plays a critical role in DNA repair, and an association between disruption of its function and increased susceptibility to cancer has been reported^14^.

*CTC1* is known causative genes of the telomere biology disorder DC, characterized by accelerated telomere shortening leading to manifestations such as bone marrow failure, cancer, and pulmonary fibrosis^15^. DC genes function in telomere maintenance; CTC1 functions in a telomere-associated complex to protect the telomere from lethal DNA degradation^16^. Although these DC genes have autosomal recessive inheritance, an association between monoallelic deleterious germline mutations and myeloid malignancies has been reported^17^.

Splice site mutations of the *SBDS*,* a causative gene of* SDS, were identified in two patients. This mutation also had relatively high frequency among the control population (0.0048 from KRGDB and 0.004 from gnomAD East Asians), probably reflecting high carrier frequency. *SBDS* is characterized by exocrine pancreatic insufficiency, skeletal abnormalities, and bone marrow failure with an increased risk of myeloid malignancy^18^. Approximately 90% of SDS is caused by two common mutations, c.183_184delinsCT and c.258 + 2T > G^19^, only the latter was detected in two of our patients. Although monoallelic mutations are not known to cause SDS, association between monoallelic mutation and increased risk of malignancy cannot be excluded.

The *DDX41* mutations were known to have different spectrum depending on ethnic groups. p.A500Cfs*9 mutation has been solely reported in Asian patients^20^. The most frequently reported mutation in Caucasians, p.D140Gfs*2, was not found in our patients. The germline *DDX41* mutation is one of most frequently detected germline predisposition mutations in myeloid malignancy, with around 70 families described to date^21^. In these families, myeloid malignancies were associated with normal karyotypes, and about 50% were found to have a somatic second hit mutation in *DDX41*, suggesting that *DDX41* acts as a tumor suppressor^22^. *DDX41* is associated with the dominant inheritance and donor cell derived leukemia is already reported^20^, screening of germline predisposition mutation should be considered during donor selection.

### Germline predisposition mutations were associated with earlier age of onset

Characteristics of patients with germline predisposition mutations are given in Table 2. There was a significant difference in age of onset between the two groups. Patients with germline predisposition mutations tended to be younger, showing an earlier age of onset (*p* = 0.005). Notably, while only 21 patients under age 20 were included (21/180, 11.7%), five among 13 with germline mutation (5/13, 38.5%) were children. Five of 21 children and eight of 139 adults had germline predisposition mutations (*p* = 0.005). Although certain mutations like those in *DDX41* are reported not to be associated with early onset malignancy^2^, age of AML diagnosis was significantly lower in patients with germline mutations in other genes such as *CEBPA*^3^. It is understandable that inherited disorders tends to be expressed at earlier age in childhood. < Considering better prognosis of children in patients with acute lymphoblastic leukemia, it cannot be ruled out harboring germline predisposition mutation was associated with better outcome > The effect of germline predisposition gene mutations needs to be further investigated in this younger age group.

**Table 2:** Characteristics of patients with germline predisposition mutation.

	Germline predisposition mutation		*p*^a^	OR
	Positive (n = 13) (%)	Negative (n = 167) (%)		
Median age	40 (range, 0–62)	51 (range, 1–85)	0.005**	0.96 (0.93–0.99)
**Genetics**				
*FLT3* ITD	2 (15.4)	27 (16.2)	0.941	0.94 (0.14–3.77)
Gene fusion	4 (30.8)	67 (40.1)	0.571	0.66 (0.14–2.5)
No. of somatic mutations	1–5	0–11	0.585	1.09 (0.79–1.44)
**Other test results**				
CD34 negative IP	5 (38.5)	61 (36.5)	0.889	1.09 (0.32–3.4)
Monocytic differentiation	4 (25.0)	35 (21.3)	0.483	1.67 (0.35–6.44)
WBC count (× 10^9^/L)	9,910	7,690	0.585	1.00 (1.00–1.00)
	(1.3–240.5)	(0.4–248.1)		
**Treatment related factors**				
First CR	12 (92.3)	143 (85.6)	0.510	2.01 (0.37–37.52)
Relapse	5 (31.3)	54 (32.9)	0.760	1.31 (0.32–4.78)
Expire	4 (25.0)	74 (45.1)	0.397	0.56 (0.12–2.11)
HSCT	7 (53.8)	88 (52.7)	0.936	1.05 (0.33–3.38)

*OR* odds ratio, *ITD* internal tandem duplication, *No* number, *IP* immunophenotype, *CR* complete remission, *HSCT* hematopoietic stem cell transplantation.

^a^Fisher’s exact test was used. **P ≤ 0.01

Notably, although the association between germline predisposition gene mutations and certain somatic mutations was not statistically clear. In addition, somatic mutations of *RUNX1* and *ASXL1* and complex karyotype, which are well-known poor prognostic factors, were not identified in the germline predisposition mutation positive group.

### Presence of germline predisposition gene mutations did not affect the clinical outcome

The presence or absence of germline predisposition mutations, however, did not affect clinical outcome. Factors confirmed as significant in this study were well-recognized good or poor prognostic factors of AML.

In multivariate Cox proportional hazards regression analysis for overall survival (OS), complex karyotype, older age, absence of gene fusion, poor outcome of induction chemotherapy, and *FLT3* ITD mutation were factors for unfavorable outcome (Supplemental Table 3). In the same analysis for relapse-free survival (RFS), poor outcome factors were *RUNX1* somatic mutation and *FLT3* ITD (Supplemental Table 4). On the other hand, complete remission after induction chemotherapy, the presence of gene fusions, and CD34-negative immunophenotype mutation were identified as favorable factors. Achievement of complete remission after induction chemotherapy and carrying well-known poor prognostic features like *FLT3* ITD mutation were important factors for both OS and RFS.

The presence or absence of germline predisposition mutations did not affect clinical outcome. As reason for the negative finding, the number of identified mutations was possibly insufficient to determine the effects of those mutations. More patients and germline mutation carriers would be needed to establish whether germline predisposition mutations are beneficial or harmful. Clinical and therapeutic heterogeneity of patients might also play the role. Comparison of patients between groups with otherwise identical condition would be desirable.

Although younger age could be linked to higher durable intensity of and better response to chemotherapy, the association of germline predisposition mutation and treatment outcome was not definite. Because statistical significance could not be achieved partially due to insufficient sample size, further study with more patients is needed.

In conclusion, we identified 13 patients with germline predisposition mutation among 180 patients with AML. Most of the mutated genes are involved in the DNA repair system, contributing genomic stability. Although the effect of these mutations on clinical outcome, including OS and RFS, was not significant, we confirmed that this group of patients tends to develop AML at a younger age. Since each mutation was rare, further study with a larger number of cases would be needed to establish the effect of the mutations.

## Supplementary information

Supplementary Information.

## References

[1] Arber DA (2016). The 2016 revision to the World Health Organization classification of myeloid neoplasms and acute leukemia. Blood.

[2] Lewinsohn M (2016). Novel germ line DDX41 mutations define families with a lower age of MDS/AML onset and lymphoid malignancies. Blood.

[3] Tawana K (2015). Disease evolution and outcomes in familial AML with germline CEBPA mutations. Blood.

[4] Bellissimo DC, Speck NA (2017). Mutations in inherited and sporadic leukemia. Front. Cell Dev. Biol..

[5] Swerdlow SH (2017). WHO Classification of Tumours of Haematopoietic and Lymphoid Tissues.

[6] Richards S (2015). Standards and guidelines for the interpretation of sequence variants: a joint consensus recommendation of the American College of Medical Genetics and Genomics and the Association for Molecular Pathology. Genet. Med..

[7] Jackson SP, Bartek J (2009). The DNA-damage response in human biology and disease. Nature.

[8] Che R, Zhang J, Nepal M, Han B, Fei P (2018). Multifaceted Fanconi anemia signaling. Trends. Genet..

[9] Ellis NA, Offit K (2012). Heterozygous mutations in DNA repair genes and hereditary breast cancer: a question of power. PLoS Genet..

[10] Cleary SP (2003). Heterozygosity for the BLM(Ash) mutation and cancer risk. Cancer Res..

[11] Berwick M (2007). Genetic heterogeneity among Fanconi anemia heterozygotes and risk of cancer. Cancer Res..

[12] Suhasini AN, Brosh RM (2012). Fanconi anemia and Bloom’s syndrome crosstalk through FANCJ–BLM helicase interaction. Trends Genet..

[13] Chan KL, Palmai-Pallag T, Ying S, Hickson ID (2009). Replication stress induces sister-chromatid bridging at fragile site loci in mitosis. Nat. Cell Biol..

[14] Lin Z (2008). A variant of the Cockayne syndrome B gene ERCC6 confers risk of lung cancer. Hum. Mutat..

[15] Kelmenson DA, Hanley M (2017). Dyskeratosis congenita. N. Engl. J. Med..

[16] Miyake Y (2009). RPA-like mammalian Ctc1-Stn1-Ten1 complex binds to single-stranded DNA and protects telomeres independently of the Pot1 pathway. Mol. Cell.

[17] Shen W (2019). Impact of germline CTC1 alterations on telomere length in acquired bone marrow failure. Br. J. Haematol..

[18] Shimamura A, Alter BP (2010). Pathophysiology and management of inherited bone marrow failure syndromes. Blood Rev..

[19] Boocock GR (2003). Mutations in SBDS are associated with Shwachman–Diamond syndrome. Nat. Genet..

[20] Kobayashi S (2017). Donor cell leukemia arising from preleukemic clones with a novel germline DDX41 mutation after allogenic hematopoietic stem cell transplantation. Leukemia.

[21] Cheah JJC, Hahn CN, Hiwase DK, Scott HS, Brown AL (2017). Myeloid neoplasms with germline DDX41 mutation. Int. J. Hematol..

[22] Polprasert C (2015). Inherited and somatic defects in DDX41 in myeloid neoplasms. Cancer Cell.

