# Supplementary material for: Prevalence and clinical implications of germline predisposition gene mutations in patients with acute myeloid leukemia
Source: Sci Rep. 2020 Aug 31;10:14297. doi: 10.1038/s41598-020-71386-z (PMC7459095; doi:10.1038/s41598-020-71386-z)
Supplement: Supplementary file 1 — Supplementary Information. [file 41598_2020_71386_MOESM1_ESM.pdf]

Prevalence and clinical implications of germline predisposition gene mutations in patients with acute myeloid leukemia

Borahm Kim[1,2], Woobin Yun[3], Seung-Tae Lee*[2], Jong Rok Choi[2], Keon HeeYoo[4], Hong Hoe Koo[4], Chul Won Jung[5], and Sun Hee Kim*[6]

[1]Department of Laboratory Medicine, Korea University College of Medicine, Seoul, Korea
[2]Department of Laboratory Medicine, Yonsei University College of Medicine, Seoul, Korea
[3]Brain Korea 21 PLUS Project for Medical Science, Yonsei University, Seoul, Korea
[4]Department of Pediatrics, Samsung Medical Center, Sungkyunkwan University School of Medicine
[5]Department of Internal Medicine, Samsung Medical Center, Sungkyunkwan University School of Medicine
[6]Department of Laboratory Medicine and Genetics, Samsung Medical Center, Sungkyunkwan University School of Medicine, Seoul, Korea

Supplemental Methods

Supplemental Tables (1-6)

Supplemental Figures (1 2)

Supplemental Methods

1. Gene panel sequencing

1) Gene panel

Gene panel sequencing was performed for 41 patients. The panel is composed of 215 genes including 113 genes known for predisposition to myeloid neoplasm.[1] All coding exons were included and intronic regions in which known pathogenic mutations according to ClinVar database (version 20170502) and HGMD professional (version 16.02) were added to the target regions.

2) Preparation and sequencing

Genomic DNA was extracted using a QIAamp DNA Blood Mini *KIT* (Qiagen, Venlo, The Netherlands). Approximately 1.5 μg of genomic DNA, extracted from bone marrow was fragmented to segments of between 150 and 250 bp using the Bioruptor Pico Sonication System (Diagenode, Belgium). The resulting DNA was then end-repaired and ligated to Illumina adapters (Illumina, San Diego, CA, USA) following the manufacturer's protocol. Sequence indexes were added to the samples to allow all samples to be sequenced in a single flow cell. Small fragments of ~100 bp and unligated adapters were removed from the mix by AMPure purification (Agencourt Bioscience, Beverly, MA, USA). Sequencing libraries were then hybridized with the probes following the manufacturer's instructions (Celemics, Seoul, Korea). Streptavidin-coated paramagnetic beads were then added and allowed to bind to the biotinylated capture probes. An external magnetic field was then applied and unbound DNA was removed. The bound captured DNA was finally eluted from the magnetic beads by digestion of the cRNA capture probes and purified. The enriched DNA was then amplified using universal primers targeting the paired-end adapters, clusters were generated, and DNA sequenced on a NextSeq 550 instrument (Illumina) with 2×151 bp reads.

3) Data analysis

Reads were aligned, using Burrows-Wheeler alignment (BWA) tool (version 0.7.12), to human genomic reference sequences (GRCh37).[2] To identify small nucleotide variations (SNVs) and indels, HaplotypeCaller and Mutect2 in the GATK package (3.8-0) and VarScan2 (2.4.0) were used and the results of the three algorithms were compared and merged.[3-5] A split-read analysis was done using Pindel (version 0.2.0) to detect large indels,

especially *FLT3* internal tandem duplication.[6] Exonic copy number variations (CNV) were analyzed using R package Exomedepth and Off target analysis was performed to assess genomic CNV using CopywriteR.[7] [8] All mutations were annotated using ANNOVAR and VEP (87) software.[9] [10] Variants were classified according to guidelines.[11] [12] Tier 1 and Tier 2 somatic variants and Pathogenic/likely pathogenic germline variants were selected for further analysis. All variants were examined by visual inspection using the Integrative Genomic Viewer.[13]

2. Whole exome sequencing

A total of 139 patients were subjected to whole exome sequencing. Genomic DNA was extracted using a QIAamp DNA Blood Mini KIT (Qiagen). The sequencing libraries for Exome-sequencing were prepared using the Twist Human Core Exome KIT (Twist Bioscience, San Francisco, CA, USA). Paired-end 100 bp read sequencing was performed on a NovaSeq system (Illumina). Alignment to human genome (NCBI build 37) and variant calling were performed using Dragen (Illumina). All mutations were annotated using ANNOVAR and VEP (87) software.[9] [10] Variants were classified according to guidelines.[11] [12] Tier 1 and Tier 2 somatic variants and Pathogenic/likely pathogenic germline variants were selected for further analysis. All variants were examined by visual inspection using the Integrative Genomic Viewer.[13]

References

1. Swerdlow SH, Campo E, Harris NL, *et al.* WHO Classification of Tumours of Haematopoietic and Lymphoid Tissues. Revised Fourth Edition. 122-128 (Lyon, 2017)
2. Li H. Toward better understanding of artifacts in variant calling from high-coverage samples. *Bioinformatics* 2014;30:2843-51.
3. Cibulskis K, Lawrence MS, Carter SL, *et al.* Sensitive detection of somatic point mutations in impure and heterogeneous cancer samples. *Nat Biotechnol* 2013;31:213-9.
4. DePristo MA, Banks E, Poplin R, *et al.* A framework for variation discovery and genotyping using next-generation DNA sequencing data. *Nat Genet* 2011;43:491-8.
5. Koboldt DC, Zhang Q, Larson DE, *et al.* VarScan 2: somatic mutation and copy number alteration discovery in cancer by exome sequencing. *Genome Res* 2012;22:568-76.
6. Ye K, Schulz MH, Long Q, *et al.* Pindel: a pattern growth approach to detect break points of large deletions and medium sized insertions from paired-end short reads. *Bioinformatics* 2009;25:2865-71.

7. Plagnol V, Curtis J, Epstein M, et al. A robust model for read count data in exome sequencing experiments and implications for copy number variant calling. *Bioinformatics* 2012;28: 2747-2754.

8. Kuilman T, Velds A, Kemper K, et al. CopywriteR: DNA copy number detection from off-target sequence data. *Genome Biol* 2015;16: 49

9. McLaren W, Pritchard B, Rios D*, et al.* Deriving the consequences of genomic variants with the Ensembl API and SNP Effect Predictor. *Bioinformatics* 2010;26:2069-70.

10. Wang K, Li M, Hakonarson H. ANNOVAR: functional annotation of genetic variants from high-throughput sequencing data. *Nucleic Acids Res* 2010;38:e164.

11. Richards S, Aziz N, Bale S, *et al*. Standards and guidelines for the interpretation of sequence variants: a joint consensus recommendation of the American College of Medical Genetics and Genomics and the Association for Molecular Pathology. *Genet Med* 2015;17: 405-424.

12. Li MM, Datto M, Duncavage EJ, *et al*. Standards and Guidelines for the Interpretation and Reporting of Sequence Variants in Cancer: A Joint Consensus Recommendation of the Association for Molecular Pathology, American Society of Clinical Oncology, and College of American Pathologists. *J Mol Diagn* 2017;19:4–23.

13 . Robinson JT, Thorvaldsdottir H, Winckler W*, et al.* Integrative genomics viewer. *Nat Biotechnol* 2011;29:24-6.

Supplemental Table 1. Clinical characteristics of patients

		No.	%
Total		180	
Median age		50.5 (0-85)	
	Adult (age >18, n=159)	52 (19-85)	
	Child (n=21)	8.5 (0-18)	
Sex	M	101	56.1
	F	79	43.9
Treatment	Chemotherapy	115	72.3
	ATRA	18	11.3
	Hypomethylating therapy	26	16.4
Hematopoietic stem cell transplantation		95	59.7
	Adult	87	91.6
	Child	8	8.4
	From a family member	54	56.8
	From an unrelated donor	33	34.7
Relapse		59	32.8
	Adult	53	
	Child	6	
Died		78	43.3
	Adult	74	
	Child	4	
First complete remission		155	86.1
	Adult	137	
	Child	18	

Supplemental Table 2. List of genes included in targeted gene panel

ABCB1, ABCB7, ABCG2, ABCG5, ABCG8, ABL1, ABL2, ACD, ACTB, ACTN1, ADA, ADAMTS13, AIRE, AK1, AK2, AKT2, ALAS2, ALDOA, AMN, ANK1, ANKRD26, AP3B1, ARID1A, ARPC1B, ASXL1, ATG2B, ATM, ATR, ATRX, AXIN1, BCL11B, BCL2, BCL6, BCOR, BCORL1, BHLHE41, BIRC3, BLM, BPGM, BRAF, BRCA1, BRCA2, BRCC3, BRINP3, BRIP1, BTG1, BTK, BTLA, C3, C4BPA, C4BPB, CALN1, CALR, CARD11, CASP10, CBL, CBLB, CBLC, CCND1, CD200, CD247, CD27, CD36, CD3D, CD3E, CD40LG, CD46, CD58, CD59, CD79B, CDAN1, CDKN1B, CDKN2A, CDKN2B, CEBPA, CFB, CFH, CFHR1, CFHR3, CFHR4, CFHR5, CFI, CHD1, CHD4, CHD9, CHMP2B, CLPB, CNOT3, COX4I2, CREBBP, CRLF2, CSF1R, CSF2RA, CSF3R, CTC1, CTCF, CTSC, CUBN, CUX1, CXCR4, CYB5R3, CYBA, CYBB, CYCS, DCLRE1C, DDX41, DGKE, DGKH, DHFR, DIS3, DKC1, DNM2, DNMT1, DNMT3A, EBF1, ECT2L, EED, EGFR, EGLN1, EGLN2, EGLN3, EHMT1, ELANE, EP300, EPAS1, EPB41, EPB42, EPCAM, EPO, EPOR, ERCC4, ERG, ETNK1, ETV6, EZH2, F2R, FANCA, FANCB, FANCC, FNACD2, FANCE, FANCF, FANCG, FNACI, FANCL, FANCM, FAS, FASLG, FAT1, FBXW7, FCGR1A, FCGR3B, FERMT3, FLI1, FLNA, FLT3, FOXP3, G6PC3, G6PD, GATA1, GATA2, GATA3, GCLC, GFI1, GFI1B, GIF, GINS1, GLRX5, GNAS, GNB1, GP1BA, GP1BB, GP9, GPI, GPRC5A, GPX1, GSKIP, GSN, GSR, GSS, HAX1, HBA1, HBA2, HBB, HBD, HCLS1, HFE, HIF1A, HIF1AN, HIF3A, HK1, HNRNPK, HOOK1, HOXA10, HOXA11, HRAS, HSPA9, HUWE1, ID3, IDH1, IDH2, IFNG, IFNGR1, IFNGR2, IKZF1, IKZF2, IKZF3, IL12RB1, IL2RB, IL2RG, IL3RA, IL7R, IRF1, ITGA2, ITGA2B, ITGB2, ITGB3, ITK, ITPKB, JAGN1, JAK1, JAK2, JAK3, JAKMIP2, JMJD1C, KDM5C, KDM6A, KDM7A, KIF23, KIT, KLF1, KMT2A, KMT2C, KMT2D, KRAS, LAMB4, LAMTOR2, LAPTM5, LCK, LEF1, LIG4, LMNA, LMO1, LMO2, LPIN2, LRP1B, LRRC4, LUC7L2, LYL1, LYST, MAD2L2, MAGT1, MAP2K1, MAP2K2, MASTL, MBL2, MECOM, MED13, MEF2B, MEF2C, MEFV, MET, MLH1, MLLT10, MLLT3, MPL, MSH2, MSH4, MSH6, MTA1, MTAP, MTR, MTRR, MVK, MYB, MYC, MYD88, MYH9, MYSM1, NAF1, NBEAL2, NBN, NCF2, NCOR2, NF1, NHEJ1, NHP2, NLRP3, NOD2, NOP10, NOTCH1, NOTCH2, NPM1, NR3C1, NRAS, NT5C2, NT5C3A, NTRK3, NUP214, OS9, P2RY2, PALB2, PARN, PAX5, PBX1, PC, PCDHB1, PDGFRA, PDGFRB, PDHA1, PDHX, PFKL, PFKM, PGK1, PGM3, PHF6, PICALM, PIEZO1, PIGA, PIK3CD, PIK3R1, PKLR, PML, PMS2, PNP, POT1, PRDM1, PRF1, PRKACG, PRPF40B, PTCH2, PTEN, PTK2B, PTPN11, PTPN2, PTPRC, PTPRD, PUS1, RAB27A, RAC1, RAC2, RAD21, RAD50, RAD51, RAD51C, RAF1, RAG1, RAG2, RB1, RBBP6, RBM8A, RELN, RFWD3, RHAG, RHOA, RIT1, RMRP, RNF168, RPL10, RPL11, RPL15, RPL23, RPL26, RPL27, RPL31, RPL35A, RPL36, RPL5, RPS10, RPS14, RPS15, RPS17, RPS19, RPS24, RPS26, RPS27, RPS27A, RPS28, RPS29, RPS7, RTEL1, RUNX1, RUNX1T1, SAMD9L, SBDS, SBF2, SEC23B, SERPING1, SETBP1, SETD2, SF1, SF3A1, SF3B1, SH2B3, SH2D1A, SHOC2, SLC11A2, SLC19A2, SLC25A38, SLC2A1, SLC35C1, SLC37A4, SLC4A1, SLCO1B1, SLCO1B3, SLFN14, SLX4, SMARCD2, SMC1A, SMC3, SOS1, SPINK5, SPRED1, SPTA1, SPTB, SRC, SRCAP, SRP72, SRSF2, STAG1, STAG2, STAT3, STAT5B, STEAP3, STX11, STXBP2, SUZ12, SYNE1, TAL1, TAL2, TAZ, TBL1XR1, TBX1, TCF3, TCIRG1, TEC, TERC, TERF1, TERF2, TERF2IP, TERT, TET1, TET2, TET3, THBD, THPO, TINF2, TLX1, TLX3, TMPRSS6, TNFAIP3, TNFRSF13B, TNFRSF14, TNFRSF1A, TOX, TP53, TPI1, TPMT, TRAF3, TRNT1, TSLP, TSR2, TUBB1, TYK2, U2AF1, U2AF2, UBE2T, UGT1A1, UGT1A7, UNC13B, UNC13D, UNC5D, USB1, USH2A, USP9X, VHL, VPS13B, VPS45, VWF, WAS, WDR1, WIPF1, WRAP53, WT1, XBP1, XIAP, XK, XRCC2, YARS2, ZAP70, ZFHX4, ZNF197, ZRSR2, MRE11A, WHSC1, STON1, OBFC1

Supplemental Table 3. List of genes included in germline predisposition genes according to WHO classification

ANKRD26, BRCA2 (FANCD1), BRIP1 (FANCJ), CEBPA, CSF3R, CTC1, DDX41, DKC1, ELANE, ETV6, FANCA, FANCB, FANCC, FANCD2, FANCE, FANCF, FANCG, FANCI, FANCL, FANCM, G6PC3, GATA1, GATA2, GFI1, HAX1, NHP2, NOP10, PALB2 (FANCN), RAD51C, RPL11, RPL35A, RPL5, RPS10, RPS17, RPS19, RPS24, RPS26, RPS7, RTEL1, RUNX1, SBDS, SLX4 (BTBD12), TERC, TERT, TlNF2, WAS

Supplemental Table 4. Factors affecting overall survival

Characteristics	p	HR	p	HR
	Univariate		Multivariate†	
Age	0.00035**	1.03 (1.01-1.04)	0.018*	1.02 (1.00-1.03)
Complete remission	0.00002**	0.33 (0.20-0.20)	0.001**	0.38 (0.22-0.67)
Gene fusion	0.00002**	0.30 (0.17-0.17)	0.023*	0.50 (0.27-0.91)
Complex karyotype	0.00635**	3.56 (1.43-1.43)	0.013*	3.38 (1.29-8.86)
FLT3 ITD	0.00758**	2.05 (1.21-1.21)	0.044*	1.84 (1.02-3.33)
ASXL1	0.024*	2.47 (1.13-1.13)	0.383	1.51 (0.60-3.81)
RUNX1	0.003**	2.48 (1.36-1.36)	0.183	1.62 (0.79-3.32)
Monocytic differentiation	0.270	1.33 (0.80-0.80)		
CD34 negative IP	0.069	0.63 (0.39-0.39)		
WBC count	0.378	1.00 (1.00-1.00)		
Platelet count	0.851	1.00 (1.00-1.00)		
Number of somatic mutation	0.042	1.12 (1.00-1.00)		
Germline mutation	0.233	0.54 (0.20-0.20)		
NPM1	0.591	1.18 (0.65-0.65)		
TP53	0.730	1.28 (0.31-0.31)		
biallelic *CEBPA*	0.135	0.22 (0.03-0.03)		
IDH	0.837	1.07 (0.55-0.55)		
TET2	0.068	1.77 (0.96-0.96)		

Abbreviations: HR, hazard ratio; IP, immunophenotype; ITD, internal tandem duplication;
Cox proportional hazard regression model was used.
†Multivariate model includes seven variables: age, complete remission, gene fusion, complex karyotype, *FLT3* ITD, *ASXL1*, and *RUNX1*

Supplemental Figure 1 Overall survival according to factors

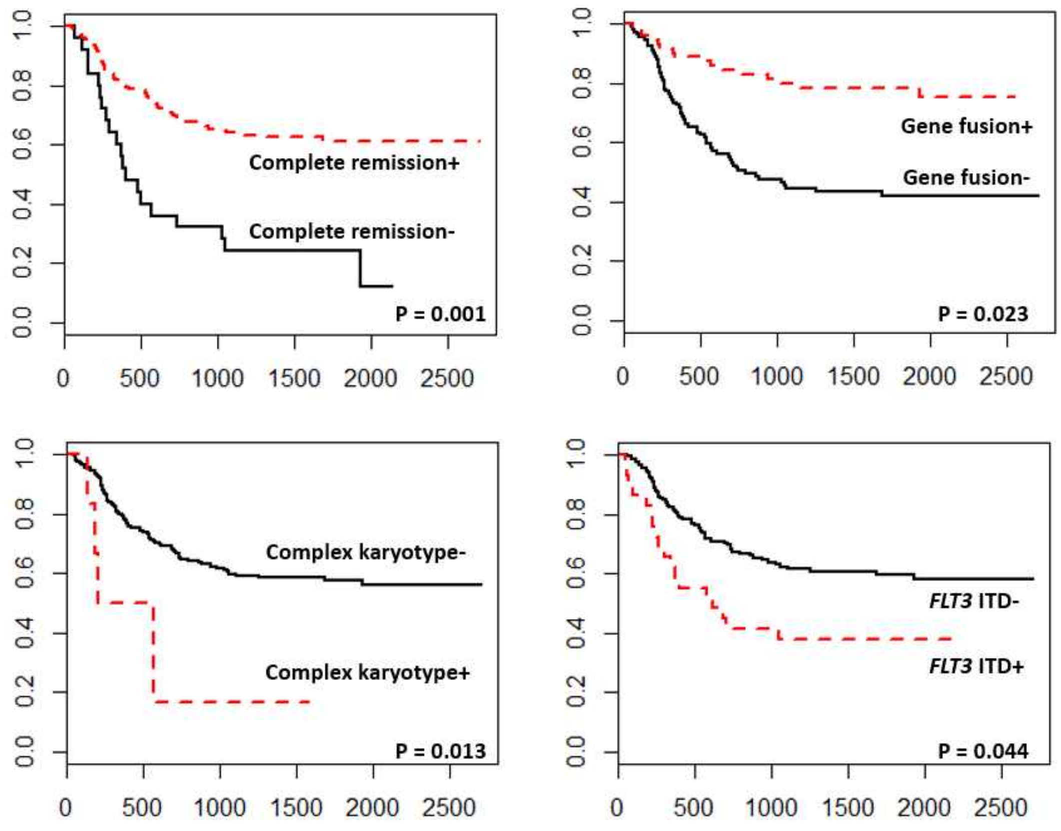

Supplemental Table 5. Factors affecting relapse free survival

Characteristics	p	HR	p	HR
	Univariate		Multivariate†	
Age	0.116	1.01 (1.00-1.00)	0.788	1.00 (0.99-1.02)
Complete remission	9.27×10^{-11}**	0.16 (0.09-0.09)	9.22×10^{-11}**	0.14 (0.08-0.25)
Gene fusion	8.43×10^{-6}**	0.20 (0.10-0.10)	0.0003**	0.24 (0.11-0.52)
Complex karyotype	0.651	1.39 (0.34-0.34)	0.662	1.39 (0.32-6.01)
FLT3 ITD	0.007**	2.33 (1.25-1.25)	0.001**	3.21 (1.59-6.48)
ASXL1	0.422	1.52 (0.55-0.55)	0.532	0.70 (0.23-2.15)
RUNX1	2.00×10^{-4}**	3.43 (1.81-1.81)	0.023*	2.38 (1.13-5.03)
CD34 negative IP	0.007**	0.43 (0.23-0.23)	0.016*	0.43 (0.21-0.85)
Monocytic differentiation	0.532	1.21 (0.66-0.66)		
WBC count	0.082	1.00 (1.00-1.00)		
Platelet count	0.980	1.00 (1.00-1.00)		
Number of somatic mutation	0.115	1.03 (0.41-0.41)		
Germline predisposition mutation	0.951	1.17 (0.59-0.59)		
NPM1	0.652	1.17 (0.59-0.59)		
TP53	0.759	0.73 (0.10-0.10)		
biallelic *CEBPA*	0.219	0.29 (0.04-0.04)		
IDH	0.890	0.95 (0.43-0.43)		
TET2	0.245	1.56 (0.74-0.74)		

Abbreviations: HR, hazard ratio; IP, immunophenotype; ITD, internal tandem duplication;
Cox proportional hazard regression model was used.
†Multivariate model includes seven variables: age, complete remission, gene fusion, complex karyotype, *FLT3* ITD, *ASXL1*, *RUNX1,* and CD34 negative IP

Supplemental Figure 2 Relapse free survival according to factors

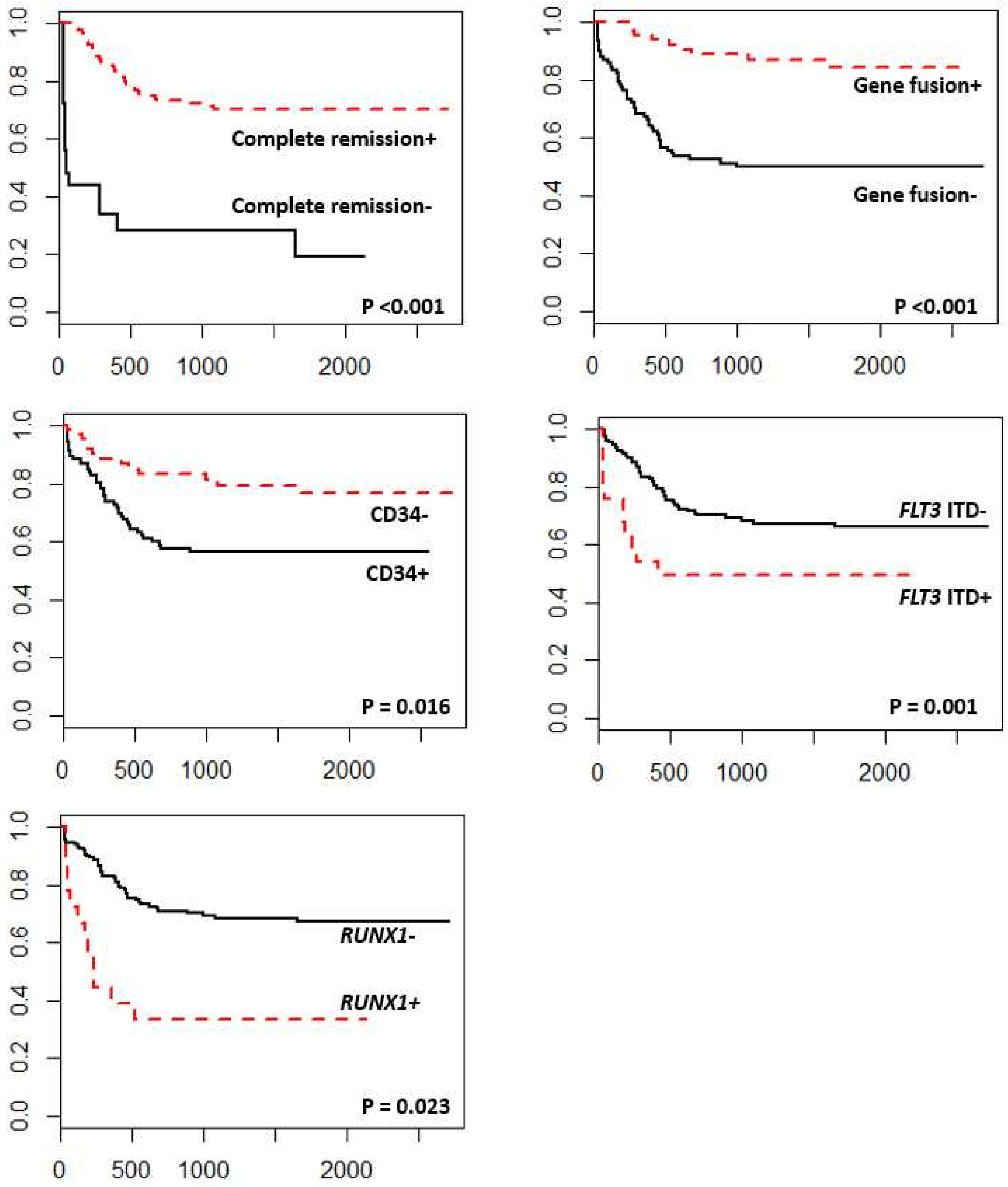

Supplemental Table 6. Selected variants identified (which can be somatic or germline origin, excluding definitely benign variants)

ID	gene	Variant			VAF
3362	NRAS	NM_002524.4	c.182A>G	p.Gln61Arg	0.39437
3362	TET2	NM_001127208.2	c.3732_3733delCT	p.Tyr1245LeufsTer22	0.45217
3362	STAG2	NM_001042749.1	c.2184+1G>T		0.9375
3362	FANCI	NM_001113378.1	c.3919G>A	p.Glu1307Lys	0.53333
1766	CEBPA	NM_004364.3	c.238dupG	p.Asp80GlyfsTer28	0.48696
1766	MLH1	NM_000249.3	c.1153C>T	p.Arg385Cys	0.5082
1766	CEBPA	NM_004364.3	c.937_939dupAAG	p.Lys313dup	0.42598
2618	CDH15	NM_004933.2	c.221delA	p.Gln74ArgfsTer47	0.54983
2618	RPS24	NM_001142285.1	c.811A>C	p.Ile271Leu	0.5582
0034	MPL	NM_005373.2	c.1775_1776delinsTTCCCGATG	p.Arg592LeufsTer23	0.25879
0034	WT1	NM_024426.4	c.1096C>T	p.Gln366Ter	0.34538
0034	CEBPA	NM_004364.3	c.150_163delGCCGCTGGGCGGCA	p.Glu50AspfsTer53	0.80488
1788	DDX41	NM_016222.2	c.1496dupC	p.Ala500CysfsTer9	0.41909
2712	BCORL1	NM_021946.4	c.1298delT	p.Val433AlafsTer11	0.27423
2712	IDH2	NM_002168.2	c.515G>A	p.Arg172Lys	0.26715
2712	BLM	NM_000057.2	c.1211G>A	p.Arg404Gln	0.50382
2712	GATA2	NM_001145661.1	c.740C>T	p.Pro247Leu	0.44444
1988					
1395					
2406	TET2	NM_001127208.2	c.4709_4710dupTG	p.Arg1571Ter	0.33846
2406	NPM1	NM_002520.6	c.860_863dupTCTG	p.Trp288CysfsTer12	0.41228
2406	TET2	NM_001127208.2	c.3353dupA	p.Asn1118LysfsTer12	0.31624
2406	DNMT3A	NM_022552.4	c.2644C>T	p.Arg882Cys	0.33
2406	FLT3	NM_004119.2	c.1752_1817dupCTCAGATAATGAGTACTTCTACGTTGATTTCAGAGAATATGAATATGATCTCAAATGGGAGTTTCC	p.Ser585_Pro606dup	0.81651
2406	FBXW7	NM_033632.3	c.692C>G	p.Pro231Arg	0.38832
3225	KIT	NM_000222.2	c.2447A>T	p.Asp816Val	0.53906
3225	SEC23B	NM_032985.4	c.155C>T	p.Pro52Leu	0.50431
3225	KMT2D	NM_003482.3	c.5977G>A	p.Gly1993Ser	0.49206
3227	NPM1	NM_002520.6	c.860_863dupTCTG	p.Trp288CysfsTer12	0.51136
3227	DNMT3A	NM_022552.4	c.2644C>T	p.Arg882Cys	0.45143
1762	RUNX1	NM_001754.4	c.561_562insGATAGAACGTTTT	p.Thr188AspfsTer29	0.40061
1762	RUNX1	NM_001754.4	c.235delG	p.Val79TrpfsTer43	0.4607
1762	BCORL1	NM_021946.4	c.2706_2707insGC	p.Thr903AlafsTer23	0.48031
1762	FLT3	NM_004119.2	c.2503G>T	p.Asp835Tyr	0.3557
1762	KCNA4	NM_002233.3	c.1913C>G	p.Thr638Arg	0.31405
1762	BCORL1	NM_021946.4	c.2707A>T	p.Thr903Ser	0.46917
1860	SUZ12	NM_015355.2	c.1615delA	p.Ser539AlafsTer13	0.31373
1860	ZRSR2	NM_005089.3	c.676G>T	p.Glu226Ter	0.94231

1860	FANCG	NM_004629.1	c.1589_1591delATA	p.Asp530_Thr531delinsAla	0.49479
3268	SUZ12	NM_015355.2	c.1850G>A	p.Trp617Ter	0.39252
3268	NPM1	NM_002520.6	c.860_863dupTCTG	p.Trp288CysfsTer12	0.47059
3268	IDH1	NM_005896.2	c.394C>T	p.Arg132Cys	0.41566
0006	CSF3R	NM_156039.3	c.2427dupC	p.Ser810GlnfsTer6	0.35827
0006	SETBP1	NM_015559.2	c.3425T>A	p.Leu1142Gln	0.46701
0006	SETBP1	NM_015559.2	c.4358G>A	p.Arg1453Lys	0.46084
0292	ATM	NM_000051.3	c.2251-10T>G		0.52239
0760	RAD50	NM_005732.3	c.326_329delCAGA	p.Thr109AsnfsTer20	0.45062
0760	CEBPA	NM_004364.3	c.478dupA	p.Ile160AsnfsTer10	0.22481
0760	ASXL1	NM_015338.5	c.1933_1934delGG	p.Gly645TrpfsTer12	0.51596
0760	RUNX1	NM_001754.4	c.1040_1041dupTG	p.His348CysfsTer247	0.38608
0760	TET2	NM_001127208.2	c.707dupA	p.Asp236GlufsTer18	0.39153
0760	BCOR	NM_001123383.1	c.1582A>T	p.Lys528Ter	0.97605
0760	TET2	NM_001127208.2	c.2368C>T	p.Gln790Ter	0.42056
0760	DNMT3A	NM_022552.4	c.2645G>C	p.Arg882Pro	0.48649
0760	KMT2A	NM_001197104.1	c.4060C>T	p.Pro1354Ser	0.41436
0760	KMT2D	NM_003482.3	c.13892C>A	p.Pro4631His	0.5914
0760	BCORL1	NM_021946.4	c.1933C>A	p.Pro645Thr	0.97041
0798					
0040					
0299	SBF2	NM_030962.3	c.2784A>T	p.Arg928Ser	0.56923
0299	TCIRG1	NM_006019.3	c.157G>A	p.Val53Ile	0.48341
0299	ATM	NM_000051.3	c.8794G>A	p.Glu2932Lys	0.4
0299	EPAS1	NM_001430.4	c.1695T>G	p.Ser565Arg	0.48372
0299	ATR	NM_001184.3	c.4606G>A	p.Val1536Ile	0.44715
0480	IDH2	NM_002168.2	c.515G>A	p.Arg172Lys	0.38202
0480	DNMT3A	NM_022552.4	c.2545C>T	p.Pro849Ser	0.43779
0780	RUNX1	NM_001754.4	c.1225_1232delGCCTCGGC	p.Ala409ArgfsTer188	0.49057
0780	FLT3	NM_004119.2	c.2503G>T	p.Asp835Tyr	0.33065
2641	MYBPC3	NM_000256.3	c.3190+5G>A		0.5942
2641	CDAN1	NM_138477.2	c.813delC	p.Thr272ProfsTer34	0.53901
2641	CUX1	NM_001913.3	c.610delG	p.Ala204LeufsTer22	0.35238
2641	BCOR	NM_001123383.1	c.3961G>A	p.Glu1321Lys	0.48819
2968	RUNX1T1	NM_175635.2	c.806C>G	p.Ala269Gly	0.47552
0001	ASXL1	NM_015338.5	c.1249C>T	p.Arg417Ter	0.44509
0001	COL5A1	NM_000093.4	c.4765G>A	p.Ala1589Thr	0.48729
0001	NRAS	NM_002524.4	c.181C>A	p.Gln61Lys	0.36041
0001	TET2	NM_001127208.2	c.3785G>A	p.Arg1262Gln	0.45614
0141	IDH1	NM_005896.2	c.395G>A	p.Arg132His	0.5
0141	FLT3	NM_004119.2	c.1784_1804dupGAGAATATGAATATGATCTCA	p.Arg595_Leu601dup	0.33
0141	SMC1A	NM_006306.3	c.2032_2034delAAG	p.Lys678del	0.44351
0141	CDKN2B	NM_078487.2	c.67G>A	p.Ala23Thr	0.46693
0141	STAG2	NM_001042749.1	c.3409A>G	p.Ser1137Gly	0.47012
0186	WT1	NM_024426.4	c.1141_1144dupTCGG	p.Ala382ValfsTer4	0.46471

0186	EPAS1	NM_001430.4	c.2069G>A	p.Arg690Gln	0.43511
0186	FANCD2	NM_001018115.1	c.911A>G	p.Lys304Arg	0.4065
0343	TET2	NM_001127208.2	c.3220_3221delCT	p.Leu1074Ter	0.42667
0343	FLT3	NM_004119.2	c.1775_1837dupTTGATTTCAGAGAATATGAATATGATCTCAAATGGGAGTTTCCAAGAGAAAATTTAGAGTTTG	p.Val592_Phe612dup	0.73333
0343	KDR	NM_002253.2	c.3071G>A	p.Cys1024Tyr	0.5283
0343	ETV6	NM_001987.4	c.1265C>A	p.Thr422Asn	0.57692
0343	ATXN7L1	NM_020725.1	c.2375T>G	p.Ile792Ser	0.52985
0381					
1025	STAG2	NM_001042749.1	c.328C>T	p.Arg110Ter	0.66667
1025	NRAS	NM_002524.4	c.35G>A	p.Gly12Asp	0.52885
1473	ASXL1	NM_015338.5	c.1276A>T	p.Lys426Ter	0.46409
1473	TET2	NM_001127208.2	c.720dupT	p.Ala241CysfsTer13	0.42
1473	TET2	NM_001127208.2	c.2083dupA	p.Met695AsnfsTer17	0.46746
1473	ZRSR2	NM_005089.3	c.122-2A>T		0.98
1716	SMC3	NM_005445.3	c.430-1G>A		0.43902
1716	DNMT3A	NM_022552.4	c.733_735delCCT	p.Pro245del	0.49606
1716	FLT3	NM_004119.2	c.1778A>G	p.Asp593Gly	0.38065
1716	SMC1A	NM_001281463.1	c.-4delT		0.99296
1906	KDM6A	NM_021140.2	c.3284+1G>A		0.30882
1906	SH2B3	NM_005475.2	c.1067_1092dupACCATTTCCTGTCCTGCTACCCCTGG	p.Phe365ThrfsTer17	0.50382
1906	CEBPA	NM_004364.3	c.90dupC	p.Phe31LeufsTer77	0.35429
1906	TET2	NM_001127208.2	c.4182+1delG		0.44444
1906	CEBPA	NM_004364.3	c.895_915delAGCCGCGACAAGGCCAAGCAG	p.Ser299_Gln305del	0.2765
1906	SMC3	NM_005445.3	c.3346T>C	p.Ser1116Pro	0.38614
2878	CBL	NM_005188.3	c.1135C>T	p.Gln379Ter	0.31579
2878	ASXL1	NM_015338.5	c.1940dupC	p.Gly649TrpfsTer9	0.38462
2878	STAG2	NM_001042749.1	c.775C>T	p.Arg259Ter	0.41436
2878	RUNX1	NM_001754.4	c.611G>T	p.Arg204Leu	0.36306
2878	U2AF1	NM_001025203.1	c.470A>C	p.Gln157Pro	0.41264
2878	ETNK1	NM_018638.4	c.724G>C	p.Ala242Pro	0.41808
2997					
3178	TET2	NM_001127208.2	c.3804-1G>C		0.56098
3178	TET2	NM_001127208.2	c.5084delG	p.Gly1695ValfsTer24	0.34783
3178	RUNX1	NM_001754.4	c.484A>G	p.Arg162Gly	0.53571
3178	RUNX1	NM_001754.4	c.677_678insAAG	p.Leu225_Ser226insArg	0.30579
0064	TET2	NM_001127208.2	c.1117C>T	p.Gln373Ter	0.4698
0064	TET2	NM_001127208.2	c.5083_5093dupGGTTATGGAAA	p.Asn1698LysfsTer25	0.46
1177	IDH2	NM_002168.2	c.419G>A	p.Arg140Gln	0.47727
1177	ETV6	NM_001987.4	c.1200_1221delCTACTACAAACTAAACATTATC	p.His400GlnfsTer14	0.27966
1177	SMC1A	NM_006306.3	c.2420G>T	p.Arg807Leu	0.93671

1177	NRAS	NM_002524.4	c.181C>A	p.Gln61Lys	0.30233
1232	ASXL1	NM_015338.5	c.2246_2249delTCCC	p.Leu749ProfsTer22	0.47039
1232	BCOR	NM_001123383.1	c.2064_2065insA	p.Gly689ArgfsTer51	0.47344
1232	ETV6	NM_001987.4	c.1195C>T	p.Arg399Cys	0.35802
1232	PARN	NM_002582.3	c.597C>G	p.Asn199Lys	0.37121
1552	FLT3	NM_004119.2	c.1767_1793dupCTTCTACGTTGATTTCAGAGAATATGA	p.Tyr597_Glu598insAspPheTyrValAspPheArgGluTyr	0.26667
2611	DNMT3A	NM_022552.4	c.1441_1460dupTACGAGGTGCGGCAGAAGTG	p.Arg488ThrfsTer170	0.43137
0302	ETV6	NM_001987.4	c.232delT	p.Ser78LeufsTer2	0.39205
0302	RUNX1	NM_001754.4	c.395_396delTG	p.Val132AspfsTer5	0.32271
0302	GPRC5A	NM_003979.3	c.80G>A	p.Trp27Ter	0.48544
0302	RUNX1	NM_001754.4	c.485G>A	p.Arg162Lys	0.46857
0427	TP53	NM_000546.5	c.743G>T	p.Arg248Leu	0.32468
0557	FLT3	NM_004119.2	c.2359G>T	p.Asp787Tyr	0.31081
0557	FANCA	NM_000135.2	c.2420C>T	p.Ala807Val	0.29655
1383					
2528	CEBPA	NM_004364.3	c.1066_1069delinsCTTGGTCAAG	p.Asn356_Cys357delinsLeuGlyGlnGly	0.25
2528	TET2	NM_001127208.2	c.859A>T	p.Lys287Ter	0.46584
2825	DNMT3A	NM_022552.4	c.2645G>A	p.Arg882His	0.40244
2825	SF3A1	NM_005877.4	c.1072-2A>G		0.30702
2825	NPM1	NM_002520.6	c.860_863dupTCTG	p.Trp288CysfsTer12	0.61314
2825	KMT2A	NM_005933.3	c.8257T>A	p.Leu2753Ile	0.44554
2924	IDH1	NM_005896.2	c.394C>T	p.Arg132Cys	0.35514
2924	BRINP3	NM_199051.1	c.833G>C	p.Cys278Ser	0.39175
2924	SRSF2	NM_003016.4	c.287C>T	p.Pro96Leu	0.38113
0016	CEBPA	NM_004364.3	c.68dupC	p.His24AlafsTer84	0.45
0016	RUNX1	NM_001754.4	c.496C>T	p.Arg166Ter	0.30065
0016	CEBPA	NM_004364.3	c.62_63insAGAGGAG	p.Ser21ArgfsTer89	0.37222
0016	ASXL1	NM_015338.5	c.2254dupG	p.Ala752GlyfsTer22	0.50282
0016	TET2	NM_001127208.2	c.651delC	p.Val218TrpfsTer32	0.43673
0016	CEBPA	NM_004364.3	c.-9_-8delAA		0.30864
0016	CUX1	NM_181552.3	c.2975C>T	p.Thr992Met	0.40891
0028	KCNQ1	NM_000218.2	c.796C>A	p.Leu266Met	0.51724
1581					
3003	AP2S1	NM_004069.3	c.44delG	p.Arg15ProfsTer17	0.36364
3003	NF1	NM_001042492.2	c.7627dupA	p.Ser2543LysfsTer3	0.24299
3003	NF1	NM_001042492.2	c.2033dupC	p.Ile679AspfsTer21	0.36095
1140					
1155	CDKN1B	NM_004064.3	c.285dupC	p.Lys96GlnfsTer29	0.33463
1155	RUNX1	NM_001754.4	c.595G>A	p.Gly199Arg	0.35096
1178					
1449	RUNX1	NM_001754.4	c.1016_1028dupTGCCCTCCATCTC	p.Asp344AlafsTer260	0.37705
1449	HNRNPK	NM_002140.3	c.869dupC	p.Pro291SerfsTer12	0.43173
1449	WT1	NM_024426.4	c.1394A>G	p.His465Arg	0.41228
1449	FLT3	NM_004119.2	c.2503G>T	p.Asp835Tyr	0.4
1449	CREBBP	NM_004380.2	c.2518G>T	p.Ala840Ser	0.48627

1832	BRIP1	NM_032043.2	c.2830C>G	p.Gln944Glu	0.52761
1832	U2AF1	NM_001025203.1	c.101C>A	p.Ser34Tyr	0.46429
2015					
2426	F12	NM_000505.3	c.405C>A	p.Cys135Ter	0.51818
2426	NPM1	NM_002520.6	c.860_863dupTCTG	p.Trp288CysfsTer12	0.38608
2453	ASXL1	NM_015338.5	c.1773C>A	p.Tyr591Ter	0.33758
2453	U2AF1	NM_001025203.1	c.470A>G	p.Gln157Arg	0.31195
2453	U2AF1	NM_001025203.1	c.101C>T	p.Ser34Phe	0.30213
2688	NRAS	NM_002524.4	c.436G>A	p.Ala146Thr	0.42963
2688	JAK1	NM_002227.2	c.2399T>C	p.Ile800Thr	0.37344
2688	LYST	NM_000081.3	c.727G>C	p.Ala243Pro	0.45294
1192	WT1	NM_024426.4	c.1104_1105insGTGT	p.Arg369ValfsTer17	0.34831
1192	FLT3	NM_004119.2	c.1822_1823insCGGATTCAGAGAATATGAATATGATCTCAAATGGGAGTTTCCAAGAG	p.Arg607_Glu608insAlaAspPheArgGluTyrGluTyrAspLeuLysTrpGluPheProArg	0.4717
1256	BLM	NM_000057.2	c.320dupT	p.Leu107PhefsTer36	0.44737
1411					
1544	SMC3	NM_005445.3	c.430-1G>C		0.32099
1544	PTEN	NM_000314.4	c.949G>A	p.Val317Ile	0.34328
1544	KCNQ4	NM_004700.3	c.140T>C	p.Leu47Pro	0.70588
1544	KIT	NM_000222.2	c.2447A>T	p.Asp816Val	0.30986
1935	IDH2	NM_002168.2	c.419G>A	p.Arg140Gln	0.5597
1935	BCOR	NM_001123383.1	c.4326+1G>A		0.96296
1935	DNMT3A	NM_022552.4	c.2645G>A	p.Arg882His	0.42857
0339	CEBPA	NM_004364.3	c.68dupC	p.His24AlafsTer84	0.51613
0339	CEBPA	NM_004364.3	c.925_930delGAGACG	p.Glu309_Thr310del	0.44444
0352	RUNX1	NM_001754.4	c.259_268dupGGCGAGCTGG	p.Val90GlyfsTer51	0.34188
0352	U2AF1	NM_001025203.1	c.101C>T	p.Ser34Phe	0.53005
0352	FLT3	NM_004119.2	c.1788_1832dupATATGAATATGATCTCAAATGGGAGTTTCCAAGAGAAAATTTAGA	p.Tyr597_Glu611dup	0.33333
0352	DDX41	NM_016222.2	c.878G>A	p.Arg293His	0.45192
0725					
0979	NCOR2	NM_006312.5	c.1531_1532insAGC	p.Gln510dup	0.34711
0979	KMT2C	NM_170606.2	c.13319A>G	p.Tyr4440Cys	0.41667
1191	KDM6A	NM_021140.2	c.3336dupC	p.Val1113ArgfsTer38	0.37013
1191	NTF4	NM_006179.4	c.617G>A	p.Arg206Gln	0.6
1191	EZH2	NM_004456.4	c.2233_2234insTTG	p.Ile744_Glu745insVal	0.443
1191	EZH2	NM_004456.4	c.1948-9_1948-8insTAGGAGT		0.40566
1233					
1647	SMARCB1	NM_003073.3	c.1121G>A	p.Arg374Gln	0.2963
1711	JAK2	NM_004972.3	c.1849G>T	p.Val617Phe	0.35802
1711	TET2	NM_001127208.2	c.3247C>T	p.Gln1083Ter	0.47739
1711	TET2	NM_001127208.2	c.2746C>T	p.Gln916Ter	0.45665
1711	FLT3	NM_004119.2	c.1789_1830dupTATGAATATGATCTCAAAT	p.Tyr597_Leu610dup	0.38182

			GGGAGTTTCCAAGAG AAAATTTA		
2014	C17orf97	NM_001013672.4	c.890C>T	p.Pro297Leu	0.63636
2404	SMC1A	NM_006306.3	c.2132G>A	p.Arg711Gln	0.92391
2404	FLT3	NM_004119.2	c.1789_1790insGGGA CGGCTCCTCAGATAA TGAGTACTTCTACGT TGATTTCAGAGAAT	p.Glu596_Tyr597ins TrpAspGlySerSerAs pAsnGluTyrPheTyr ValAspPheArgGlu	0.73846
2938	WT1	NM_024426.4	c.1322dupA	p.His441GlnfsTer14	0.51471
2938	ERCC4	NM_005236.2	c.1043A>T	p.Asp348Val	0.4717
2938	FBXW7	NM_033632.3	c.268G>A	p.Asp90Asn	0.41111
0674	FLT3	NM_004119.2	c.2359G>T	p.Asp787Tyr	0.39623
0878	NCOR2	NM_006312.5	c.1531_1532insAGCAG C	p.Gln509_Gln510du p	0.54854
1364	FLRT2	NM_013231.4	c.656A>C	p.Lys219Thr	0.54225
1781	EP300	NM_001429.3	c.6488C>G	p.Pro2163Arg	0.44286
2124	F8	NM_000132.3	c.3637delA	p.Ile1213PhefsTer5	1
2124	RUNX1	NM_001754.4	c.781_782delCC	p.Pro261SerfsTer33 8	0.43966
2124	NPM1	NM_002520.6	c.772-1G>C		0.34
2124	FLT3	NM_004119.2	c.1752_1805dupCTCA GATAATGAGTACTTC TACGTTGATTTCAGA GAATATGAATATGAT CTCAA	p.Leu601_Lys602in sAsnSerAspAsnGlu TyrPheTyrValAspPh eArgGluTyrGluTyrA spLeu	0.52273
2124	RTEL1	NM_032957.4	c.3115C>A	p.Gln1039Lys	0.50442
1834	CEBPA	NM_004364.3	c.335_343delinsG	p.Pro112Argfs*55	0.32609
1834	TET2	NM_001127208.2	c.2646C>A	p.Cys882Ter	0.33628
1834	RAD21	NM_006265.2	c.1820_1823dupAAGC	p.Ile609SerfsTer3	0.42857
1834	XIAP	NM_001167.3	c.1048_1050delGAG	p.Glu350del	1
1834	CEBPA	NM_004364.3	c.898C>G	p.Arg300Gly	0.37171
2578	WT1	NM_024426.4	c.1381_1388delTCCCG GTC	p.Ser461ArgfsTer13	0.5
2578	CEBPA	NM_004364.3	c.209delC	p.Pro70ArgfsTer90	0.43889
2709					
2717					
2759	RPS24	NM_001142285.1	c.853T>C	p.Ser285Pro	0.43721
2759	PALB2	NM_024675.3	c.909_911delCCT	p.Leu304del	0.42411
2759	ATRX	NM_000489.3	c.1825C>G	p.Pro609Ala	0.98925
2856	C17orf97	NM_001013672.4	c.1017_1018insAA	p.Glu340LysfsTer79	0.34061
2856	CHEK2	NM_007194.3	c.1111C>T	p.His371Tyr	0.39516
2931					
2975					
3036	SBDS	NM_016038.2	c.258+2T>C		0.51049
3036	NRAS	NM_002524.4	c.34G>A	p.Gly12Ser	0.41584
3036	XIAP	NM_001167.3	c.1048_1050delGAG	p.Glu350del	0.45455
3076					
3090	LDLR	NM_000527.4	c.344G>A	p.Arg115His	0.43969
3090	FLT3	NM_004119.2	c.1820_1821insGAGTG AATATGATCTCAAAT GGGAGTTTCCAAG	p.Arg607_Glu608ins SerGluTyrAspLeuLy sTrpGluPheProArg	0.46809
0104	IDH2	NM_002168.2	c.419G>A	p.Arg140Gln	0.42956
0104	NPM1	NM_002520.6	c.860_863dupTCTG	p.Trp288CysfsTer12	0.44099

0104	BRCA2	NM_000059.3	c.3487G>C	p.Asp1163His	0.5
0104	CDH1	NM_004360.3	c.2494G>A	p.Val832Met	0.51175
0379	ASXL1	NM_015338.5	c.1946_1947insGAAG	p.Gly650LysfsTer9	0.44014
0392	NPM1	NM_002520.6	c.860_863dupTCTG	p.Trp288CysfsTer12	0.51479
0392	TET2	NM_001127208.2	c.3626T>G	p.Leu1209Arg	0.95808
0392	DDX41	NM_016222.2	c.98A>G	p.Tyr33Cys	0.44785
0413					
0481	DNMT3A	NM_022552.4	c.1464delG	p.Asn489ThrfsTer162	0.44275
0481	IDH2	NM_002168.2	c.515G>A	p.Arg172Lys	0.44215
0502					
0617	MYBPC3	NM_000256.3	c.2504G>T	p.Arg835Leu	0.4433
0617	GATA2	NM_001145661.1	c.1078T>C	p.Trp360Arg	0.37349
0617	JAK3	NM_000215.3	c.1503G>T	p.Gln501His	0.4411
0788					
0878	FLT3	NM_004119.2	c.1755_1837+1dupAGATAATGAGTACTTCTACGTTGATTTCAGAGAATATGAATATGATCTCAAATGGGAGTTTCCAAGAGAAAATTTAGAGTTTGG		0.77019
0878	TET2	NM_001127208.2	c.5693C>T	p.Ser1898Phe	0.95623
1281	PTPN11	NM_002834.3	c.1472C>T	p.Pro491Leu	0.5298
1281	TET2	NM_001127208.2	c.3894dupT	p.Lys1299Ter	0.94634
1281	NPM1	NM_002520.6	c.860_863dupTCTG	p.Trp288CysfsTer12	0.3125
1281	LUC7L2	NM_001244585.1	c.735_739delAGAGA	p.Glu246ArgfsTer37	0.34444
1281	FLT3	NM_004119.2	c.2503G>A	p.Asp835Asn	0.32353
1353	FLT3	NM_004119.2	c.1803_1804insCGCGGCTCCTCAGATAATGAGTACTTCTACGTTGATTTCAGAGAATATGAATATGATCTC	p.Leu601_Lys602insArgGlySerSerAspAsnGluTyrPheTyrValAspPheArgGluTyrGluTyrAspLeu	0.32386
1353	DCLRE1C	NM_001033855.1	c.1150G>A	p.Asp384Asn	0.47059
1657	ASXL1	NM_015338.5	c.2423delC	p.Pro808LeufsTer10	0.36181
1657	RUNX1	NM_001754.4	c.497G>A	p.Arg166Gln	0.62105
1716					
1820	STAG2	NM_001042749.1	c.1068T>A	p.Tyr356Ter	0.37931
2065	NRAS	NM_002524.4	c.35G>A	p.Gly12Asp	0.28095
2065	NPM1	NM_002520.6	c.863_864insCGCG	p.Trp288CysfsTer12	0.41379
2065	IDH1	NM_005896.2	c.395G>A	p.Arg132His	0.30159
2065	NRAS	NM_002524.4	c.34G>A	p.Gly12Ser	0.11
2117	IDH2	NM_002168.2	c.419G>A	p.Arg140Gln	0.45815
2117	DNMT3A	NM_022552.4	c.2645G>A	p.Arg882His	0.55556
2117	NPM1	NM_002520.6	c.860_863dupTCTG	p.Trp288CysfsTer12	0.49419
2117	BRINP3	NM_199051.1	c.623C>A	p.Thr208Lys	0.52128
2133	CEBPA	NM_004364.3	c.80_86delGCAGCGC	p.Ser27ThrfsTer131	0.32161
2133	CEBPA	NM_004364.3	c.866G>C	p.Arg289Pro	0.33862
2138	IDH2	NM_002168.2	c.419G>A	p.Arg140Gln	0.35738
2138	NPM1	NM_002520.6	c.863_864insCATG	p.Trp288CysfsTer12	0.34807
2138	CBL	NM_005188.3	c.1151G>A	p.Cys384Tyr	0.5988
2138	EZH2	NM_004456.4	c.1688C>T	p.Pro563Leu	0.27228

2154					
2154	CHD7	NM_017780.3	c.6184C>T	p.Arg2062Trp	0.35811
2349	WT1	NM_024426.4	c.1368T>A	p.Cys456Ter	0.36391
2349	HNF1B	NM_000458.2	c.1395C>G	p.Ser465Arg	0.42683
2349	FLT3	NM_004119.2	c.2503G>T	p.Asp835Tyr	0.32624
2457	CEBPA	NM_004364.3	c.107_114delGCGCGGGC	p.Gly36AlafsTer69	0.35593
2457	SMC1A	NM_006306.3	c.1756C>T	p.Arg586Trp	0.25862
2457	CEBPA	NM_004364.3	c.921_926delCGTGGA	p.Asn307_Glu309delinsLys	0.35242
2462	RUNX1	NM_001754.4	c.496C>T	p.Arg166Ter	0.44231
2462	SERPINC1	NM_000488.3	c.442T>C	p.Ser148Pro	0.57297
2462	U2AF1	NM_001025203.1	c.101C>T	p.Ser34Phe	0.42487
2768	RAD21	NM_006265.2	c.548T>G	p.Leu183Ter	0.416
2768	TTN	NM_001267550.1	c.160G>A	p.Val54Met	0.46909
2768	FANCA	NM_000135.2	c.2778+10C>T		0.45263
2768	FLT3	NM_004119.2	c.1743_1817dupGACCGGCTCCTCAGATAATGAGTACTTCTACGTTGATTTCAGAGAATATGAATATGATCTCAAATGGGAGTTTCC	p.Thr582_Pro606dup	0.58278
2912	FANCI	NM_001113378.1	c.158-2A>G		0.53982
2912	HNRNPK	NM_002140.3	c.512G>C	p.Arg171Pro	0.37634
2147	TP53	NM_000546.5	c.659A>G	p.Tyr220Cys	0.70874
2841	DNMT3A	NM_022552.4	c.750_751insG	p.Thr251AspfsTer2	0.40435
2841	TET2	NM_001127208.2	c.4708_4709delAT	p.Met1570GlufsTer7	0.45098
2841	NPM1	NM_002520.6	c.863_864insCCTG	p.Trp288CysfsTer12	0.51948
2841	FLT3	NM_004119.2	c.1757_1801dupATAATGAGTACTTCTACGTTGATTTCAGAGAATATGAATATGATC	p.Asp600_Leu601insHisAsnGluTyrPheTyrValAspPheArgGluTyrGluTyrAsp	0.72115
0283	BRCA1	NM_007294.3	c.427G>A	p.Glu143Lys	0.46243
0283	SMC1A	NM_006306.3	c.1756C>T	p.Arg586Trp	0.26106
0368	KRAS	NM_004985.3	c.32_37dupCTGGTG	p.Ala11_Gly12dup	0.32432
0502	MPL	NM_005373.2	c.1069C>T	p.Arg357Ter	0.41636
0502	TNNT2	NM_001001430.2	c.422G>A	p.Arg141Gln	0.54696
0502	FLT3	NM_004119.2	c.2503G>T	p.Asp835Tyr	0.3
0654	ERCC6	NM_000124.3	c.2169+1G>A		0.54128
0654	CEBPA	NM_004364.3	c.891dupC	p.Lys298GlnfsTer23	0.43946
0654	GATA2	NM_001145661.1	c.1114G>A	p.Ala372Thr	0.32143
0654	KMT2D	NM_003482.3	c.13633G>T	p.Val4545Phe	0.45188
0654	SH2B3	NM_005475.2	c.369G>C	p.Glu123Asp	0.4058
0655	SBDS	NM_016038.2	c.258+2T>C		0.53333
0655	GATA1	NM_002049.3	c.-19-1G>A		0.6
0664	WT1	NM_024426.4	c.1098+1G>A		0.89116
0738	ASXL1	NM_015338.5	c.2129delG	p.Gly710GlufsTer15	0.4502
0738	RUNX1	NM_001754.4	c.495_496insGCCGGGT	p.Arg166AlafsTer49	0.25581
0738	RUNX1	NM_001754.4	c.153dupG	p.Met52AspfsTer86	0.58746
0738	EZH2	NM_004456.4	c.1648_1649delAA	p.Lys550ValfsTer5	0.41875
0738	BCORL1	NM_021946.4	c.4107dupC	p.Ser1370LeufsTer34	0.62416

0738	EZH2	NM_004456.4	c.1979G>A	p.Gly660Glu	0.41622
1190	FLT3	NM_004119.2	c.1786_1818dupGAATATGAATATGATCTCAAATGGGAGTTTCCA	p.Glu596_Pro606dup	0.42051
1238	FANCA	NM_000135.2	c.4232C>T	p.Pro1411Leu	0.35897
1238	FLT3	NM_004119.2	c.1772_1837dupACGTTGATTTCAGAGAATATGAATATGATCTCAAATGGGAGTTTCCAAGAGAAAATTTAGAGTTTG	p.Phe612_Gly613insAspValAspPheArgGluTyrGluTyrAspLeuLysTrpGluPheProArgGluAsnLeuGluPhe	0.42177
1415	FANCI	NM_001113378.1	c.158-2A>G		0.6
1415	KIT	NM_000222.2	c.2447A>T	p.Asp816Val	0.31792
1527	BRCA2	NM_000059.3	c.9285C>T	p.Asp3095=	0.47899
1527	FANCM	NM_020937.2	c.1456C>T	p.Arg486Ter	0.53543
1538	CDH1	NM_004360.3	c.76G>C	p.Glu26Gln	0.4
1538	KIT	NM_000222.2	c.154G>A	p.Asp52Asn	0.5051
1538	FANCL	NM_001114636.1	c.21C>A	p.Ser7Arg	0.37762
1538	SF3A1	NM_005877.4	c.1418G>A	p.Arg473His	0.30952
1561	BRCA1	NM_007294.3	c.2433delC	p.Lys812ArgfsTer3	0.54331
1735	RUNX1	NM_001754.4	c.735dupC	p.Thr246HisfsTer15	0.4329
1735	MN1	NM_002430.2	c.3875_3876dupCC	p.Lys1293ProfsTer28	0.34603
1735	BCOR	NM_001123383.1	c.1798C>T	p.Gln600Ter	0.90909
1735	SCN1A	NM_006920.4	c.4753C>T	p.Arg1585Cys	0.41714
1735	RUNX1	NM_001754.4	c.400G>A	p.Ala134Thr	0.4
1790					
1834					
1852	RUNX1T1	NM_175635.2	c.1376A>G	p.Gln459Arg	0.44891
2093	WT1	NM_024426.4	c.1109delG	p.Arg370LeufsTer5	0.3806
2093	NPM1	NM_002520.6	c.863_864insCCTG	p.Trp288CysfsTer12	0.50877
2093	RAD21	NM_006265.2	c.1756C>T	p.Arg586Ter	0.49123
2093	FLT3	NM_004119.2	c.1773_1790dupCGTTGATTTCAGAGAATA	p.Val592_Tyr597dup	0.32237
2206	DNMT3A	NM_022552.4	c.2645G>A	p.Arg882His	0.44397
2206	IDH1	NM_005896.2	c.394C>T	p.Arg132Cys	0.45327
2295	CDKN1B	NM_004064.3	c.206C>T	p.Pro69Leu	0.46721
2295	FANCL	NM_001114636.1	c.268C>G	p.Leu90Val	0.35385
2347	DNMT3A	NM_022552.4	c.2029_2036delCACCAGGG	p.His677GlufsTer33	0.46875
2347	NPM1	NM_002520.6	c.860_863dupTCTG	p.Trp288CysfsTer12	0.45213
2347	SMC1A	NM_006306.3	c.1193G>A	p.Arg398Gln	0.51712
2347	PKP3	NM_007183.2	c.1715C>T	p.Pro572Leu	0.42188
2347	FLT3	NM_004119.2	c.2039C>T	p.Ala680Val	0.38298
3065	BRCA2	NM_000059.3	c.2798_2799delCA	p.Thr933ArgfsTer2	0.38686
3065	IDH2	NM_002168.2	c.419G>A	p.Arg140Gln	0.44882
3065	DNMT3A	NM_022552.4	c.2645G>A	p.Arg882His	0.40278
3065	CTNNA1	NM_001903.2	c.25A>G	p.Ile9Val	0.53846
